# Epidemiological and clinical profile of malaria patients in Isfahan Province, Iran: a retrospective analysis from 2009 to 2025

**DOI:** 10.1186/s12936-025-05735-6

**Published:** 2025-12-26

**Authors:** Sara Lesani, Mohammad Javad Boozhmehrani, Mahnaz Golban, Zahra Ghayour Najafabadi

**Affiliations:** 1 Department of Parasitology and Mycology, School of Medicine, Isfahan University of Medical Sciences, Isfahan, Iran; 2 Department of Medical Parasitology, Faculty of Medicine, Ahvaz Jundishapur University of Medical Sciences, Ahvaz, Iran; 3 Student Research Committee, Ahvaz Jundishapur University of Medical Sciences, Ahvaz, Iran

**Keywords:** Malaria, *Plasmodium vivax*, *Plasmodium falciparum*, Epidemiology, Isfahan Province, Imported cases, Elimination

## Abstract

**Background:**

Malaria remains a significant global health challenge despite remarkable declines in incidence, in Isfahan Province, Iran, historically considered an endemic area. Although national elimination programmes have reduced transmission, imported cases continue to sustain malaria risk. This study aimed to assess the epidemiological and clinical characteristics of malaria in Isfahan Province from 2009 to 2025.

**Methods:**

A retrospective observational analysis was conducted using surveillance data from the Isfahan Center for Disease Control. All microscopically confirmed malaria cases reported between January 2009 and January 2025 were included. Demographic, epidemiological, and clinical data were extracted via a standardized checklist. Descriptive statistics and multivariable logistic regression were applied to identify trends and risk factors for severe malaria.

**Results:**

A total of 569 cases were reported during the study period, with incidence declining sharply after 2009, but fluctuating during subsequent years, including a resurgence in 2024. Most cases occurred in males (96.1%) and individuals aged 15–24 years (53.6%), with Afghan nationals comprising 80.8% of infections. Imported cases represented 80.8% of the total burden, underscoring migration-related risks. *Plasmodium vivax* accounted for 88.0% of cases, while *Plasmodium falciparum* (4.6%) was strongly associated with severe disease (adjusted odds ratio 22.6; 95% CI 1.24–410.8; p = 0.035). Seasonal peaks were observed in spring and summer, and per capita incidence was higher in rural counties despite absolute urban predominance.

**Conclusions:**

Malaria incidence in Isfahan Province has markedly declined over the past 16 years; however, imported cases, predominantly among migrant workers, remain the central challenge to elimination. The dominance of *P. vivax* alongside the clinical severity of *P. falciparum* highlights the need for species-specific strategies. Strengthened cross-border collaboration, targeted interventions for migrant populations, and enhanced surveillance in high-risk rural areas are essential to sustain elimination efforts.

**Graphical abstract:**

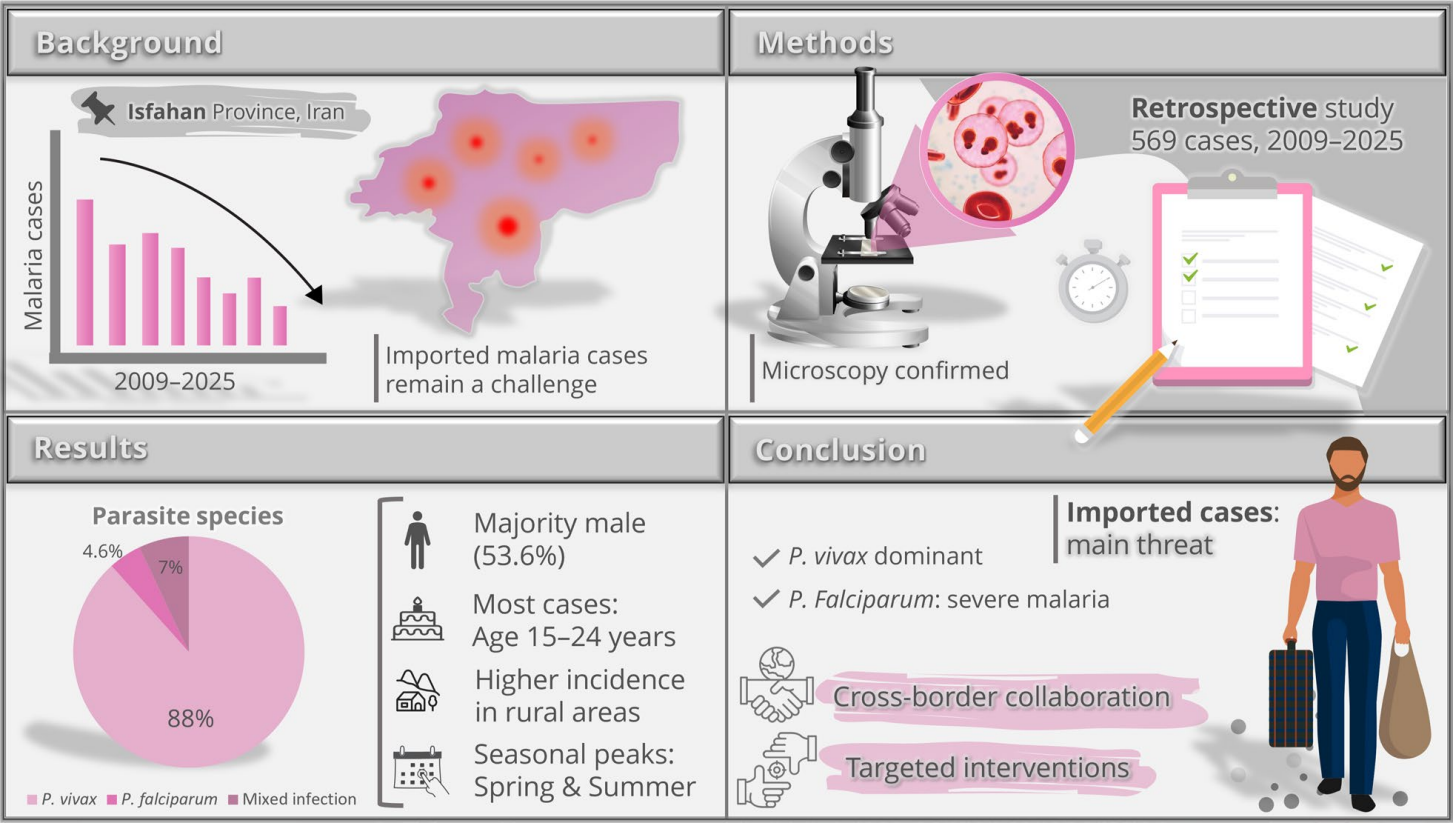

## Background

Malaria remains one of the most devastating infectious diseases worldwide, and based on the latest statistics, it was responsible for about 263 million cases and approximately 597,000 deaths worldwide in 2023 [1]. In Iran, substantial progress has been achieved in reducing the malaria burden over recent decades through the implementation of robust control strategies [2]. Nevertheless, the disease persists in certain regions, including Isfahan Province, necessitating ongoing surveillance and targeted interventions [3]. Geographically, regions between 64°N and 32°S latitude provide favourable conditions for the life cycle of the malaria parasite and its *Anopheles* mosquito vector, with the city of Isfahan situated within this zone. Historically, Isfahan has experienced periodic malaria epidemics and is recognized as an endemic area. The region's unique climatic conditions, coupled with the presence of the Zayandeh-Rud River and surrounding rice paddies, create suitable breeding grounds for *Anopheles* mosquitoes. According to reports from the Center for Disease Control, more than 700 malaria cases were documented in the province between 2004 and 2009, including both locally transmitted and imported cases [3]. Prior studies indicate that 91% of malaria cases in Isfahan are imported, originating from areas outside the province [4].

Epidemiologically, Iran is divided into three zones based on indicators such as annual parasite incidence, vector status, transmission patterns, and reported statistics: the northern Zagros Mountains region, the southern and southwestern region, and the southeastern region. Isfahan Province lies north of the Zagros Mountains and falls within the first epidemiological zone. As a major industrial hub, the province attracts diverse economic opportunities and tourism, drawing large numbers of job seekers from various parts of the country as well as foreign nationals from neighbouring countries like Afghanistan and Pakistan, with migration trends continuing steadily [4]. Recent data reveal that 3000 to 8000 malaria cases enter Iran annually from adjacent nations [5].

The predominant malaria species in Iran is *P. vivax* [6], a pattern mirrored in Isfahan, where 93.5% of patients are infected with this species [3]. Although malaria incidence has declined dramatically compared to pre-elimination eras, local transmission and outbreaks persist. A particular concern is the recent resurgence of *P. falciparum* malaria, primarily affecting migrant workers from Afghanistan.

Given the significant morbidity and mortality associated with *Plasmodium* infections, a deeper understanding of epidemiological patterns, high-risk areas, populations, and determinants of ongoing transmission in Isfahan Province is essential. Enhanced surveillance and evidence-based measures are critical to advancing malaria elimination goals in the region. This study analyzes the epidemiology of malaria in Isfahan Province between 2009 and 2025, based on surveillance data from the Center for Disease Control and the Malaria Control Group.

## Methods

### Study design and setting

This retrospective observational study analyzed the epidemiological and clinical characteristics of malaria cases in Isfahan Province, Iran. The study encompassed all reported malaria infections from January 1, 2009, to January 31, 2025. Data were sourced from the provincial Center for Disease Control and Prevention, which maintains comprehensive surveillance records on infectious diseases, including malaria.

### Participants and inclusion criteria

All individuals diagnosed with malaria during the study period were included. Diagnosis was confirmed through microscopic examination of peripheral blood smears, following standard protocols recommended by the World Health Organization (WHO) and Iran's National Malaria Control Programme. Cases were identified based on positive detection of *Plasmodium* parasites in blood smears, regardless of symptom severity or treatment outcome. No exclusion criteria were applied, ensuring a complete representation of reported cases in the province.

### Data collection

A standardized checklist was developed to extract relevant information from malaria surveillance records and patient files. The checklist encompassed demographic, epidemiological, and clinical variables, providing a comprehensive profile of affected individuals. Data were collected anonymously to ensure confidentiality, with each case assigned a unique identifier. Demographic and epidemiological characteristics included age group, sex, nationality, place of residence, season of diagnosis, and epidemiological classification (Table 1). Clinical and parasitological variables included parasite species and stage, disease severity, and treatment outcome (Table 2). Data extraction was conducted by trained research personnel to ensure accuracy and completeness. Any missing or ambiguous entries were cross-verified against the original records where possible.

**Table 1:** Demographic and epidemiological characteristics of malaria cases in Isfahan Province, 2009–2025

Characteristic	Total (n, %)
Age groups (years)	
0–4 (Early childhood)	5 (0.9)
5–14 (School-aged children)	14 (2.5)
15–24 (Youth)	305 (53.6)
25–44 (Young adults)	210 (36.9)
45–64 (Middle-aged adults)	33 (5.8)
> 65 (Older adults)	2 (0.4)
Gender	
Male	547 (96.1)
Female	22 (3.9)
Nationality	
Iranian	84 (14.8)
Afghan	460 (80.8)
Pakistani	9 (1.6)
Other	14 (2.5)
Missing	2 (0.4)
Season	
Spring	202 (35.5)
Summer	210 (36.9)
Autumn	98 (17.2)
Winter	59 (10.4)
Epidemiological classification	
Imported case	460 (80.8)
Introduced case	23 (4.0)
Relapse case / Recrudescent case	30 (5.3)
Indigenous case	37 (6.5)
Missing	19 (3.3)
Place of residence	
Rural	97 (17.0)
Urban	460 (80.8)
Missing	12 (2.1)

**Table 2:** Clinical and parasitological characteristics of malaria cases

Variable	Total (n, %)
Parasite species	
*P. vivax*	501 (88.0)
*P. falciparum*	26 (4.6)
Mixed infection	40 (7.0)
Missing	2 (0.4)
Parasite stage	
Trophozoite	207 (36.4)
Schizont	6 (1.1)
Gametocyte	7 (1.2)
Mixed	137 (24.1)
Missing	212 (37.3)
Disease severity	
Outpatient (Mild)	534 (93.8)
Hospitalized (Severe)	35 (6.2)
Treatment failure	
Yes	3 (0.5)
No	405 (71.2)
Missing	161 (28.3)

### Statistical analysis

**Fig. 1:**
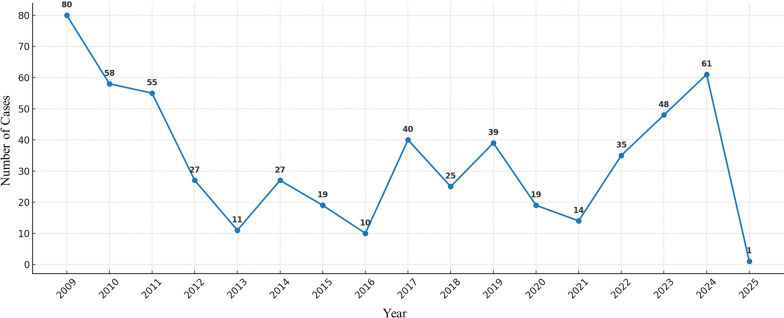
Annual malaria cases reported in Isfahan Province between 2009 and 2025. The line chart illustrates the temporal trend of confirmed cases, showing fluctuations with a general decline in earlier years followed by a resurgence in recent years

Data were analysed using SPSS software version 27.0.1. Categorical variables were summarized as frequencies and percentages. Months of diagnosis were grouped into four seasons to facilitate interpretation of temporal patterns. Time trends were assessed by plotting annual distributions of malaria cases (Fig. 1). Multivariable logistic regression was applied to identify factors associated with severe malaria, adjusting for relevant covariates. A p-value of < 0.05 was considered statistically significant. All analyses were conducted with attention to the retrospective nature of the dataset.

### Ethical considerations

The study protocol was approved by the Ethics Committee of Isfahan University of Medical Sciences (approval number: IR.ARI.MUI.REC.1403.005). As a retrospective analysis of anonymized surveillance data, individual informed consent was not required. All procedures adhered to the principles outlined in the Declaration of Helsinki and Iran's national guidelines for health research.

## Results

A total of 569 patients with malaria were identified between 2009 and early 2025, with the highest number of reported cases occurring in 2009 (n = 80; Fig. 1). The mean age of the patients was 25.5 ± 9.8 years, with ages ranging from less than 1 year to 70 years. Demographic and epidemiological characteristics of the patients are summarized in Table 1. Accounting for missing data (24.6%), 63.4% of patients were employed as workers, while 12.0% held other occupations, including guards, housewives, students, farmers, military personnel, drivers, office employees, shopkeepers, and various others.

**Fig. 2:**
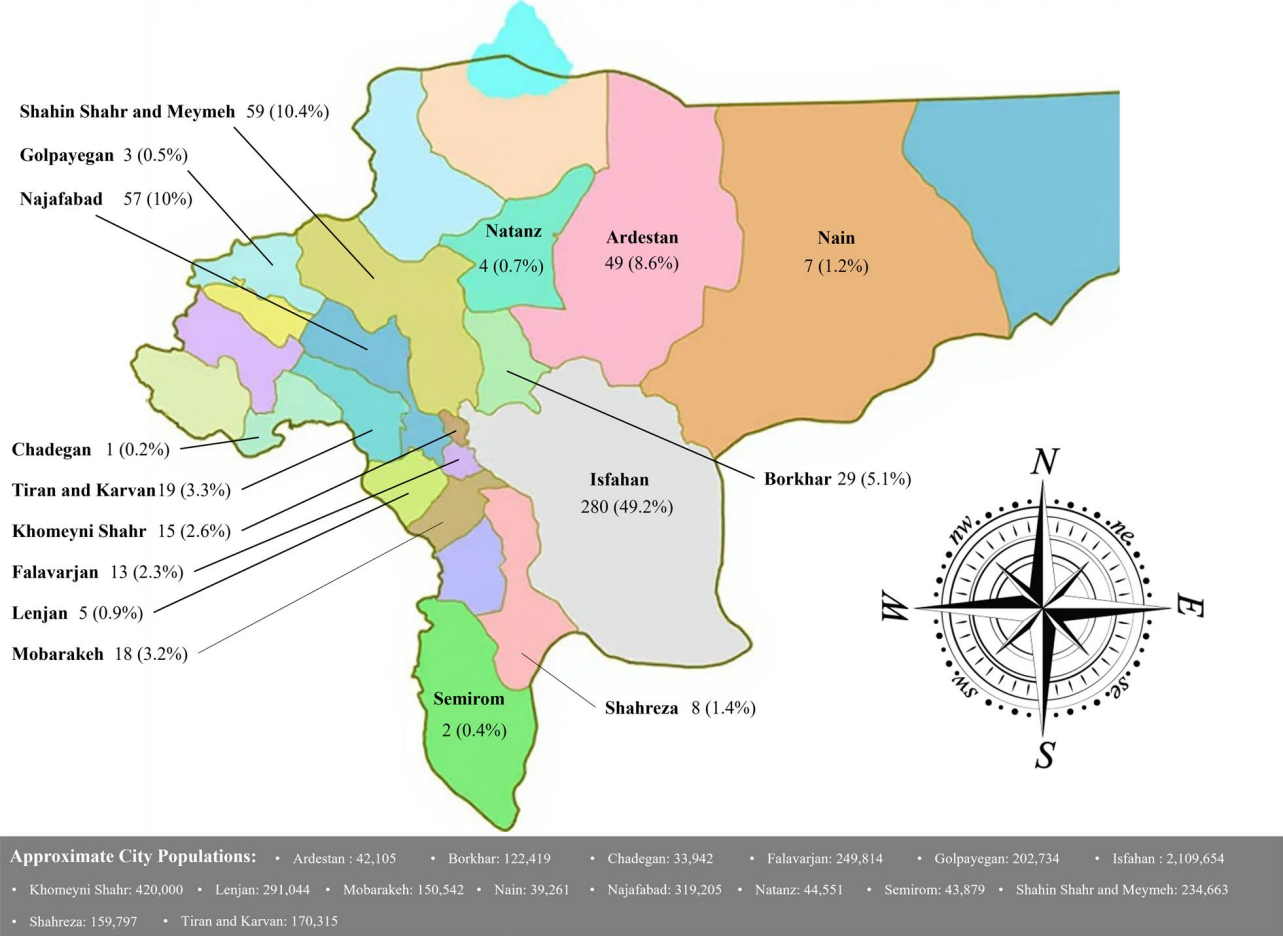
Geographic distribution of malaria cases across counties of Isfahan Province. The map shows the number and percentage of malaria cases detected in each county (relative to total provincial cases). Percentages are not adjusted for population size. Approximate population figures for each county, based on the most recent census, are provided below the map

In the multivariable logistic regression model, only parasite species demonstrated a significant association with disease severity. Infection with *P. falciparum* compared to *P. vivax* significantly increased the risk of severe disease (adjusted odds ratio, 22.6; 95% confidence interval, 1.24–410.8; P = 0.035). Other variables, including age, gender, nationality, place of residence, epidemiological classification, and treatment failure, showed no significant associations with disease severity. All cases of *P. falciparum* infection occurred in males, whereas mixed infections were only found in two women. *P. falciparum* cases were more frequent among Iranian nationals than Afghan nationals (13 vs. 12). Clinical and parasitological features are presented in Table 2. The distribution of case frequencies across cities in the province is illustrated in Fig. 2.

Among the 40 patients with mixed *Plasmodium* infections, 38 (95.0%) were male and 2 (5.0%) were female. The mean age was 28.5 ± 13.0 years, comparable to the overall patient population. Most of these infections were imported (n = 32), followed by introduced (n = 1), relapse/recrudescent (n = 2), and indigenous cases (n = 4), with one case missing classification data. Thirty-four patients (85.0%) resided in urban areas and four (10.0%) in rural areas, with two records missing. No treatment failures were observed among mixed infections, although data were incomplete for five individuals.

## Discussion

The findings of this retrospective analysis reveal a marked reduction in malaria incidence in Isfahan Province over the study period, with a total of 569 cases documented from 2009 to early 2025. The peak incidence in 2009, with 80 cases, aligns with broader national trends in Iran, where malaria cases were more prevalent before intensified elimination efforts. Nationally, malaria incidence declined from 0.24 per 1000 population in 2002 to 0.01 per 1000 in 2017, reflecting successful control measures such as enhanced surveillance and vector management [7]. Similarly, in Isfahan, the sharp decline post-2009, reaching a low of 11 cases in 2013, underscores the impact of these strategies, though intermittent peaks in 2014, 2017, and 2019 suggest vulnerabilities to outbreaks, akin to fluctuations observed in Sistan and Baluchestan Province, where malaria transmission remains endemic due to environmental and cross-border factors [7].

The predominance of *P. vivax* (88.0% of cases) in Isfahan mirrors national patterns, where *P. vivax* accounted for the majority of infections during 2002–2017, comprising over 80% in southern and southeastern provinces [7]. This species distribution is similar to regions like Pakistan and Afghanistan, where *P. vivax* is also predominant (typically accounting for 70–90% of cases), while both *P. vivax* and *P. falciparum* coexist [8, 9], but imported cases in Iran often introduce *P. falciparum*, as seen in 4.6% of Isfahan infections. Notably, the multivariable logistic regression identified *P. falciparum* as the sole significant predictor of severe disease (adjusted odds ratio 22.6; 95% CI 1.24–410.8; P = 0.035), consistent with global evidence that *P. falciparum* infections are more likely to progress to complications such as cerebral malaria or acute respiratory distress compared to *P. vivax*. In Pakistan, severe vivax malaria has been reported [10], but in Iran, including Isfahan, *P. vivax* typically manifests mildly, with 93.8% of cases managed outpatient, aligning with studies in low-transmission settings where immunity develops rapidly against this species. Regarding parasite stage, trophozoites were the most frequently observed form (36.4%), followed by mixed stages (24.1%), a distribution broadly consistent with reports from other Iranian provinces where trophozoites predominate during routine microscopic diagnosis [11]. Within the analysed dataset, mixed infections (7.0%) predominantly affected young male migrant workers and reflected the same epidemiological pattern as single-species infections, indicating that co-infections did not exhibit distinct demographic or clinical trends.

Demographic patterns in Isfahan, with 96.1% of cases in males and 53.6% in the 15–24 age group, reflect occupational and migratory risks, as 63.4% of patients were workers, often migrants. This echoes epidemiological data from Iran (2002–2017), where over 70% of cases occurred in males aged 15 and older, predominantly labourers in rural areas [7]. Afghan nationals comprised 80.8% of cases in Isfahan, highlighting the role of migration from endemic neighbours; nationally, imported cases surged from 2009, with 57.05% of infections (2011–2014) attributed to cross-border travel, primarily from Afghanistan and Pakistan [11]. In Larestan (southern Iran), 2006–2018 data showed a similar predominance of imported cases among Afghan and Pakistani migrants, emphasizing shared vulnerabilities in socioeconomically challenged border regions [12].

Epidemiologically, 80.8% of cases in Isfahan Province were classified as imported, underscoring the significant influence of cross-border migration on malaria persistence in this low-transmission setting. This high proportion of imported infections aligns with historical trends in the region, where studies have consistently highlighted the role of migrant populations from endemic neighbouring countries. For example, a prior investigation in Isfahan from 2004 to 2009 found that 91% of cases were among Afghan immigrants, with an additional 5.6% attributed to Pakistani nationals, emphasizing the importation dynamics driven by labour migration to industrial and agricultural hubs [3]. Similarly, national overviews indicate that imported cases constitute a major challenge in non-endemic provinces like Isfahan, often originating from southeastern Iran or abroad, and contributing to sporadic local transmission [7, 11]. Although 80.8% of patients reported urban residence, this figure likely reflects diagnostic and referral biases, as major healthcare centers are concentrated in urban areas, leading to underrepresentation of rural cases in absolute counts. However, population-adjusted analyses reveal a stark urban–rural disparity, with higher per capita incidence rates in smaller, more rural counties, pointing to elevated transmission risks in these areas due to factors such as agricultural practices, irrigation systems, and proximity to natural vector breeding sites like rivers and rice paddies. The three counties exhibiting the highest per capita rates included Ardestan (approximately 116 cases per 100,000 population), Shahin Shahr and Meymeh (25 per 100,000), and Najafabad (18 per 100,000), where environmental conditions and occupational exposures may facilitate mosquito proliferation and human-vector contact. In contrast, the three counties with the lowest per capita statistics were Chadegan, Semirom, and Natanz, each recording fewer than 10 cases per 100,000, potentially due to lower population densities, reduced migration inflows, or more effective local surveillance. Seasonal peaks in spring and summer (72.4% combined) correlate with vector activity, consistent with national reports of increased transmission during warmer months [13].

The observed decline during the COVID-19 pandemic (2019–2021), from 39 cases in 2019 to 14 in 2021, likely resulted from travel restrictions and hygiene measures, mirroring a national drop in imported cases [14]. However, the post-pandemic resurgence, which rose to 61 cases in 2024, highlights renewed mobility risks, as seen in northern Iran's 2023 surge of 22 imported cases in Golestan Province, linked to cross-border movements and regional outbreaks driven partly by catastrophic floods in neighbouring countries [15]. This resurgence underscores challenges in sustaining elimination, with treatment failure in 0.5% of Isfahan cases potentially signaling emerging resistance, though chloroquine remains effective against *P. vivax* nationally [16].

These patterns emphasize the need for targeted interventions in migrant populations and high-risk counties, integrating enhanced border surveillance and evidence-based measures to achieve malaria elimination goals by 2025. The persistence of imported cases, despite dramatic declines, necessitates cross-border collaboration to mitigate reintroduction risks.

## Limitations

This study has several limitations that should be acknowledged. One major limitation concerns the incomplete data on treatment completion, which were unavailable for 161 patients (28.3%). This gap restricts the ability to accurately evaluate treatment outcomes and raises the possibility that some cases classified as relapse or recrudescence may, in fact, reflect incomplete treatment or unrecorded follow-up. Although no treatment failure was documented among mixed infections, missing post-treatment data could have masked the true recurrence rate. Strengthening the recording of treatment completion and post-therapy follow-up within the national malaria surveillance system would substantially improve the accuracy of future epidemiological and therapeutic assessments.

Missing data for other demographic and epidemiological variables in some patient records may also have introduced bias, as these omissions likely resulted from incomplete documentation or inconsistencies in routine reporting. Furthermore, while Kashan and Aran–Bidgol are geographically part of Isfahan Province, their malaria data are collected and reported independently, and access to those records was not available. This limitation may have led to a slight underestimation of the province’s total malaria burden. Additionally, the retrospective design of the study restricts causal inference and is inherently susceptible to potential recording or reporting errors in archival data. Moreover, data on patients’ prior history of malaria infection were not available, preventing assessment of previous exposure or its influence on relapse or re-infection patterns. Finally, the findings are specific to the studied population and geographic context and should be interpreted cautiously when generalizing to other regions with differing ecological or socio-demographic characteristics.

## Conclusion

This retrospective analysis highlights the substantial progress toward malaria control in Isfahan Province, marked by a steady decline in incidence over the past 16 years. Nevertheless, the persistence of imported cases, predominantly among migrant workers, remains the central obstacle to elimination. The epidemiological dominance of *P. vivax* alongside the clinical severity associated with *P. falciparum* reinforces the need for species-specific management strategies. Seasonal, geographic, and demographic variations further underline the importance of localized interventions. Sustained elimination will depend on strengthening surveillance in high-risk rural areas, expanding cross-border cooperation, and tailoring public health measures to vulnerable migrant populations.

## Data Availability

The data that support the findings of this research are available from the corresponding author, Zahra Ghayour Najafabadi, upon reasonable request.

## Abbreviations

*P. vivax*: *Plasmodium vivax*
*P. falciparum*: *Plasmodium falciparum*
WHO: World Health Organization
COVID-19: Coronavirus disease 2019
SPSS: Statistical package for the social sciences
CI: Confidence interval
IR.ARI.MUI.REC: Isfahan University of Medical Sciences Ethics Committee Approval Code Prefix
